# Synthesis and Fungicidal Activity of Lansiumamide A and B and Their Derivatives

**DOI:** 10.3390/molecules23071499

**Published:** 2018-06-21

**Authors:** Huiyou Xu, Ting Chen, Luanbin Huang, Qiuju Shen, Zengwei Lian, Yan Shi, Ming-An Ouyang, Liyan Song

**Affiliations:** Key Laboratory of Biopesticide and Chemical Biology, Ministry of Education, Fujian Agriculture and Forestry University, Fuzhou 350002, China; huiyouxu@126.com (H.X.); fafuchenting@163.com (T.C.); h17746075243@163.com (L.H.); m18759775477@163.com (Q.S); fafuzengweilian@126.com (Z.L.); shiyanshelly@163.com (Y.S.); minganouyang@163.com (M.-A.O.)

**Keywords:** antifungal activity, Lansiumamide B, derivatives, SAR, chemical synthesis

## Abstract

A efficient 2-step protocol has been applied for the synthesis of Lansiumamide B (*N*-methyl-*N*-*cis*-styryl-cinnamamide, **2**) derivatives by various substitution on the amide nitrogen with alkyl, allyl, propargyl, benzyl or ester groups. The structures of nine new compounds were characterized by HRMS, ^1^H NMR, and ^13^C NMR spectra. These compounds were tested in vitro against 10 strains of phytopathogenic fungi and showed a wide antifungal spectrum. The relationship between different substituents on the amide nitrogen and antifungal activity of Lansiumamide B derivatives were compared and analyzed. The result indicates that the length and steric hindrance of *N*-substitution have a significant impact on biological activities. It is noteworthy that the methyl or ethyl substituent on the amide nitrogen is critical for the antifungal activities.

## 1. Introduction

Lansiumamide B (*N*-methyl-*N*-*cis*-styryl-cinnamamide, **2**) was firstly isolated from the extracts from *Clausena lansium* (Lour.) Skeels by Lin in 1989 [1]. Since then, Lansiumamide B has been reported to exhibit many pharmacological effects including anti-obesity [2], anti-diabetic [3], anti-necrosis [4] and anti-inflammatory [5] bioactivities. Moreover, Lansiumamide B was also evaluated as agrochemical. Intriguingly, Lansiumamide B performs a wide spectrum of efficient bioactivities, such as antifeedant activities and cytotoxicity against *Spodoptera litura* [6], anti-bacterial activity against *Ralstonia solanacearum* [7], larvicidal activity against mosquito [8], nematicidal activity against *Bursaphelenehus xylophilus* [9,10], insecticidal activity against *Plutella xylostella* and *Mythimna separata*, acaricidal activity against *Tetranychus cinnabarinus*, herbicidal activity against *Echinochloa crusgalli* and *Abutilon theophrasti* [11] and fungicidal activity against *Colletotrichun gloeosporioides*, *Fusarium oxysporum* f.sp. *cubense* [12] and *Botrytis cinerea* [11]. The biological activities of Lansiumamide B were nicely reviewed by Wan in 2012 [13].

Structurally, Lansiumamide B (**2**) was reported as a Z-configuration enamide, which is crucial to its biological activities [11] and might pose significant synthetic challenges. The combination of the potent bioactivities and the structural novelty has received considerable synthetic attention. The pioneering works of Taylor [14], Maier [15] and Fürstner [16] furnished the enamide unit as a mixture of E/Z isomers. In 2010, Gooβen [17] documented the stereoselective and one-step synthesis of Lansiumamide A through the use of a ruthenium-catalysed hydroamination of phenylacetylene and cinnamamide. In 2014, Marquez [18] developed another synthetic route to afford Lansiumamide A (**1**) as a single double bond isomer in four steps and 20% overall yield. Then NaH-mediated methylation on the amide nitrogen simply provided the desired Lansiumamide B.

Phytopathogenic fungi causes severe losses in agricultural production. Thus, the efficient control of plant fungal disease is of great importance for food production worldwide [19]. The commercially available fungicides have been extensively used in currant agriculture and lead to the growing resistance of phytopathogenic fungi [20]. And the discovery and development of new antifungal agents would be necessary to provide new arsenals for the battle against phyto-fungal disease. Natural product derived agrochemicals have received considerable attention from researchers owing to their higher efficiency, lower mammalian toxicity, and environmental compatibility [21].

**Figure 1:**
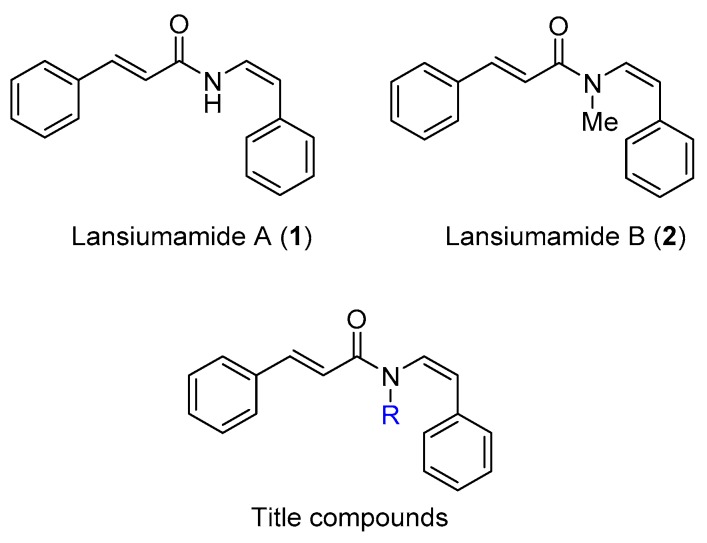
Lead compounds and design strategy for the title compounds.

Lansiumamide B is proven to be a promising lead compound for the development of novel antifungal agents [11,12]. Some Lansiumamide B-based derivatives were designed and synthesized by Wan and coworkers [22]. However, most of them showed lower antifungal activities against *Colletotrichum gloeosporioides* than the lead compound (Lansiumamide B). Thus, the Z-enamide moiety was further proven to be essential for the antifungal activities [22]. These results prompted us to figure out the structure-activity relationship (SAR) of Lansiumamide B derivatives and extend the modification of Lansiumamide B with the aim of securing more potent antifungal agents. Herein, we report a new series of Lansiumamide B derivatives containing various amide nitrogen substitutions (Figure 1) and their antifungal activities. The SAR between the title compounds and their lead compound (Lansiumamide B) is also compared and discussed. 

## 2. Results and Discussion

### 2.1. Chemical Syntheses

**Scheme 1:**
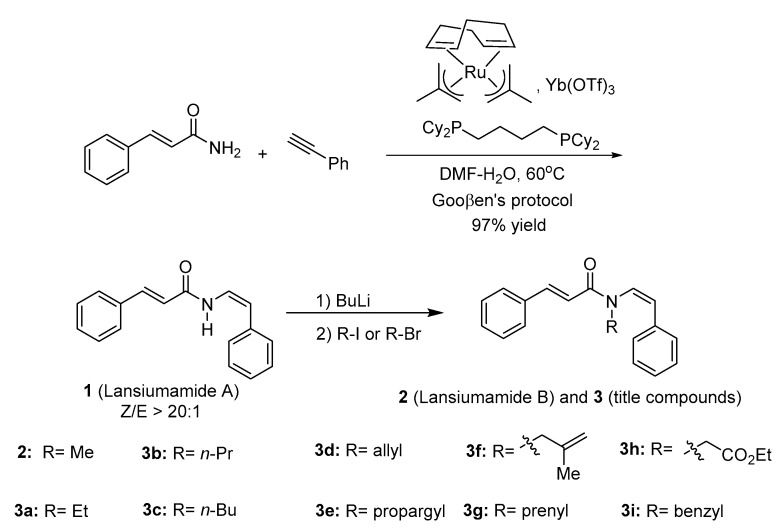
General synthetic procedure for title compounds.

The synthetic route of compounds **1**, **2** and **3** is outlined in Scheme 1. Ruthenium-catalyzed hydroamination of commercially available cinnamamide and phenylacetylene according to Gooβen's protocol gave Lansiumamide A (**1**) in a single step and an almost quantitative yield. Final BuLi-mediated substitution on the N-H moiety of Lansiumamide A (**1**) afforded Lansiumamide B (**2**) and its derivatives **3** in good to excellent yields (73% to 89%). The synthetic route is shown in Scheme 1. The structures of **3** were firmly established by HRMS, ^1^H NMR, and ^13^C NMR spectra. 

### 2.2. Fugicidal Activity

The in vitro antifungal activities of compounds **1**, **2**, **3a** to **3i** were screened against ten plant pathogenic fungi at 100 µg/mL. The results listed in Table 1 and Table 2 show all the compounds display the activity in varying degrees against each of the test fungi. Although part or most of the synthetic compounds are less active than their lead compound (Lansiumamide B), some structure-activity relationships can still be discovered. Firstly, the amide N-H moiety is detrimental to the bioactivities, which could be avoided by substitution on the amide nitrogen. Secondly, increasing the substituent from methyl to ethyl group would decrease the antifungal activity slightly. There are exceptions and it becomes more efficient to *Thanatephorus cucumeris*, *Fusarium solani*, and *Alternaria solani*. As the substituent continues to increase from the propyl to butyl group, the corresponding biological activities descend gradually. Furthermore, we notice that as the substituent in compounds **3d**, **3f**, **3g**, **3i** grows bulkier, the antifungal activity comes down rapidly. Thirdly, similar steric hindrance would result in similar inhibitory rate. For example, the *n*-propyl and allyl group come to almost the same antifungal activities, as does the propargyl group except that it becomes more powerful against *Botrytis cinerea* and *Fusahum graminearum* than the ethyl group. Finally, the ester group would not help to elevate the antifungal activity. Noteworthy is that all of our synthetic compounds exhibit much higher inhibitory activity against *Alternaria solani* than the positive control carbendazim.

## 3. Experimental Section

### 3.1. General Information

Reactions were carried out in oven or flame-dried glassware under a nitrogen atmosphere, unless otherwise noted. Tetrahydrofuran (THF) was freshly distilled before use from sodium using benzophenone as indicator. Dichloromethane was freshly distilled before use from calcium hydride (CaH_2_). All other anhydrous solvents were dried over 3 Å or 4 Å molecular sieves. Solvents used in workup, extraction and column chromatography were used as received from commercial suppliers without prior purification. Reactions were magnetically stirred and monitored by thin layer chromatography (TLC, 0.25 mm) on Liangchen pre-coated silica gel plates. Flash chromatography was performed with silica gel 60 (particle size 0.040–0.062 mm) supplied by Liangchen. ^1^H and ^13^C NMR spectra were recorded on a Bruker AVIII-400 spectrometer (400 MHz for ^1^H, 100 MHz for ^13^C) (Bruker BioSpin, Rheinstetten, Germany). Chemical shifts are reported in parts per million (ppm) as values relative to the internal chloroform (7.26 ppm for ^1^H and 77.16 ppm for ^13^C). Abbreviations for signal coupling are as follows: s, singlet; d, doublet; t, triplet; q, quartet; m, multiplet. High resolution mass spectra were measured at Keecloud Mass Spectrometry Service Company (Shanghai, China) on either a Thermo Scientific LTQ Orbitrap XL system or a Bruker solariX System. 

### 3.2. Synthetic Procedures

#### 3.2.1. General Synthetic Procedure for Lansiumamide A (**1**)

Lansiumamide A (**1**) was synthesized according to Gooβen’s protocol [17]. An oven-dried flask was charged with the *trans*-cinnamamide (147 mg, 1.00 mmol), bis(2-methallyl)-cycloocta-1,5-diene-ruthenium(II) (16.0 mg, 0.05 mmol), 1,4-bis(dicyclohexylphosphinobutane (27.0 mg, 0.06 mmol), and ytterbium triflate (24.8 mg, 0.04 mmol) and flushed with nitrogen. Subsequently, dry DMF (3 mL), phenylacetylene (204 mg, 2.00 mmol), and water (108 μL, 6.00 mmol) were added via syringe. The resulting solution was stirred for 6 h at 60 °C. The reaction mixtures were poured into aq NaHCO_3_ (30 mL). The resulting mixture was extracted with EtOAc (3 × 20 mL), the combined organic layers were washed with water (20 mL) and brine (20 mL), dried over Na_2_SO_4_, and concentrated under reduced pressure. The residue was purified by flash chromatography on silica gel using eluents (petroleum ether/ethyl acetate = 5/1) to give the Lansiumamide A (**1**) (241 mg, 0.97 mmol) as a light yellow solid. Note: reaction on a larger scale (>1 mmol) resulted in lower yield. Therefore, the reaction on a 1.0 mmol scale was repeated five times to obtain 1.20 grams of Lansiumamide A (**1**). ^1^H NMR (400 MHz, CDCl_3_) δ: 7.79 (d, *J* = 9.2 Hz, 1H), 7.73 (d, *J* = 15.6 Hz, 1H), 7.38–7.30 (m, 10H), 7.13 (dd, *J* = 10.4, 9.2 Hz, 1H), 6.37 (d, *J* = 15.6 Hz, 1H), 5.83 (d, *J* = 10.4 Hz, 1H). ^13^C NMR (100 MHz, CDCl_3_) δ: 163.22, 143.13, 135.77, 134.49, 130.19, 129.18 (2 × C), 128.91 (2 × C), 128.07 (2 × C), 128.02 (2 × C), 127.06, 122.32, 119.49, 110.72. 

#### 3.2.2. General Synthetic Procedure for Lansiumamide B (**2**) the Title Compounds **3**

A flame-dried round bottle under nitrogen atmosphere was charged with Lansiumamide A (100 mg, 0.4 mmol) and anhydrous tetrahydrofuran (THF, 2 mL). The solution was cooled to −78 °C and *n*-butyllithium (0.25 mL, 1.6 M in hexane, 0.4 mmol) was added dropwise. The reaction mixture was warmed to 0 °C, stirred for additional 30 min and then cooled to −78 °C. The corresponding iodide or bromide (0.6 mmol) was added dropwise to the cold solution. The reaction mixture was then allowed to gradually warm to room temperature and stirred overnight. Aqueous saturated NH_4_Cl solution (5 mL) was added under vigorous stirring to quench the reaction. The volatiles (mainly THF) were removed under reduced pressure and the aqueous phase was extracted with diethyl ether (3 × 10 mL). The combined organic fractions were washed with water and brine, dried over anhydrous MgSO_4_ and evaporated under reduced pressure. The residue was purified by flash column chromatography on silica gel using eluents (petroleum ether/ethyl acetate = 10/1) to afford the desired compound. The copies of ^1^H NMR and ^13^C NMR could be found in the Supplementary Materials.

**2**. 85 mg, 81% yield; light yellow solid. ^1^H NMR (400 MHz, CDCl_3_) δ: 7.67 (d, *J* = 15.6 Hz, 1H), 7.39–7.31 (m, 10H), 6.96 (d, *J* = 15.6 Hz, 1H), 6.53 (d, *J* = 8.8 Hz, 1H), 6.27 (d, *J* = 8.8 Hz, 1H), 3.12 (s, 3H). ^13^C NMR (100 MHz, CDCl_3_) δ: 166.45, 142.70, 135.17, 134.42, 129.72, 128.85, 128.74 (2 × C), 128.68 (4 × C), 128.10, 127.95 (2 × C), 125.08, 118.30, 34.67. 

**3a**. 92 mg, 83% yield; light yellow oil. ^1^H NMR (400 MHz, CDCl_3_) δ: 7.58 (d, *J* = 15.6 Hz, 1H), 7.32–7.24 (m, 10H), 6.89 (d, *J* = 15.6 Hz, 1H), 6.34 (s, 2H), 3.65 (q, *J* = 7.2 Hz, 2H), 1.20 (t, *J* = 7.2 Hz, 3H). ^13^C NMR (100 MHz, CDCl_3_) δ: 165.73, 142.44, 135.30, 134.32, 129.54, 128.67 (2 × C), 128.64 (2 × C), 128.59 (3 × C), 128.22, 127.86 (2 × C), 127.14, 118.84, 41.92, 13.15. HRMS (ESI) *m*/*z* calculated for C_19_H_20_NO [M + H]^+^ 278.1545, found 278.1550.

**3b.** 91 mg, 78% yield; light yellow oil. ^1^H NMR (400 MHz, CDCl_3_) δ: 7.59 (d, *J* = 15.6 Hz, 1H), 7.36–7.28 (m, 10H), 6.91 (d, *J* = 15.6 Hz, 1H), 6.36 (d, *J* = 8.8 Hz, 1H), 6.31 (d, *J* = 8.8 Hz, 1H), 3.55 (t, *J* = 7.6 Hz, 2H), 1.66 (dq, *J* = 14.8, 7.6 Hz, 2H), 0.90 (t, *J* = 7.2 Hz, 3H). ^13^C NMR (100 MHz, CDCl_3_) δ: 165.85, 142.47, 135.31, 134.29, 129.55, 128.68 (2 × C), 128.65 (2 × C), 128.61 (2 × C), 128.22, 127.87 (2 × C), 127.68, 126.89, 118.73, 48.85, 21.31, 11.59. HRMS (ESI) *m*/*z* calculated for C_20_H_22_NO [M + H]^+^ 292.1701, found 292.1697.

**3c**. 92 mg, 75% yield; light yellow oil. ^1^H NMR (400 MHz, CDCl_3_) δ: 7.59 (d, *J* = 15.6 Hz, 1H), 7.34–7.26 (m, 10H), 6.90 (d, *J* = 15.6 Hz, 1H), 6.36 (d, *J* = 8.8 Hz, 1H), 6.31 (d, *J* = 8.8 Hz, 1H), 3.58 (t, *J* = 7.6 Hz, 1H), 1.61 (dt, *J* = 14.8, 7.6 Hz, 2H), 1.32 (dq, *J* = 14.8, 7.2 Hz, 2H), 0.90 (t, *J* = 7.2 Hz, 3H). ^13^C NMR (400 MHz, CDCl_3_) δ: 165.82, 142.45, 135.31 (2 × C), 129.54, 128.68 (2 × C), 128.65 (2 × C), 128.60 (2 × C), 128.21, 127.87 (2 × C), 127.68, 126.84, 118.79, 46.96, 30.12, 20.36, 13.84. HRMS (ESI) *m*/*z* calculated for C_21_H_24_NO [M + H]^+^ 306.1858, found 306.1855.

**3d**. 103 mg, 89% yield; light yellow oil. ^1^H NMR (400 MHz, CDCl_3_) δ: 7.61 (d, *J* = 15.6 Hz, 1H), 7.34–7.26 (m, 10H), 6.91 (d, *J* = 15.6 Hz, 1H), 6.37 (d, *J* = 10.0 Hz, 1H), 6.32 (d, *J* = 10.0 Hz, 1H), 5.87 (ddt, *J* = 10.0, 8.0, 5.6, 1H), 5.14 (dd, *J* = 10.0, 8.0 Hz, 2H), 4.21 (d, *J* = 5.6 Hz, 2H). ^13^C NMR (100 MHz, CDCl_3_) δ: 165.81, 142.93, 135.20, 134.29, 132.78, 129.67, 128.72 (2 × C), 128.69 (2 × C), 128.61 (2 × C), 128.29, 127.92 (2 × C), 127.17, 127.05, 118.45, 117.84, 49.45. HRMS (ESI) *m*/*z* calculated for C_20_H_19_NNaO [M + Na]^+^ 312.1364, found 312.1365.

**3e**. 95 mg, 83% yield; light yellow oil. ^1^H NMR (400 MHz, CDCl_3_) δ: 7.61 (d, *J* = 15.6 Hz, 1H), 7.33–7.24 (m, 10H), 6.86 (d, *J* = 15.6 Hz, 1H), 6.42 (s, 2H), 4.39 (s, 2H), 2.23 (s, 1H). ^13^C NMR (100 MHz, CDCl_3_) δ: 165.75, 143.53, 135.03, 134.04, 129.83, 128.80 (2 × C), 128.71 (2 × C), 128.54 (2 × C), 128.46, 128.03, 127.98 (2 × C), 126.48, 117.91, 78.73, 71.98, 35.82. HRMS (ESI) *m*/*z* calculated for C_20_H_18_NO [M + H]^+^ 288.1388, found 288.1383.

**3f**. 99 mg, 82% yield; light yellow oil. ^1^H NMR (400 MHz, CDCl_3_) δ: 7.64 (d, *J* = 15.6 Hz, 1H), 7.35–7.26 (m, 10H), 6.96 (d, *J* = 15.6 Hz, 1H), 6.37 (d, *J* = 8.8 Hz, 1H), 6.32 (d, *J* = 8.8 Hz, 1H), 4.87 (s, 1H), 4.74 (s, 1H), 4.18 (s, 2H), 1.71 (s, 3H). ^13^C NMR (100 MHz, CDCl_3_) δ: 165.97, 143.12, 140.38, 135.19, 134.29, 129.70, 128.72 (2 × C), 128.70 (2 × C), 128.64 (2 × C), 128.27, 127.94 (2 × C), 127.20, 126.93, 118.30, 112.68, 52.05, 20.40. HRMS (ESI) *m*/*z* calculated for C_21_H_22_NO [M + H]^+^ 304.1701, found 304.1704.

**3g**. 100 mg, 79% yield; light yellow oil. ^1^H NMR (400 MHz, CDCl_3_) δ: 7.58 (d, J = 15.2 Hz, 1H), 7.34–7.25 (m, 10H), 6.88 (d, *J* = 15.2 Hz, 1H), 6.32 (s, 2H), 5.28 (t, *J* = 7.2 Hz, 1H), 4.21 (d, *J* = 7.2 Hz, 2H), 1.69 (s, 3H), 1.62 (s, 3H). ^13^C NMR (100 MHz, CDCl_3_) δ: 165.70, 142.47, 136.54, 135.32, 134.43, 129.53, 128.65 (4 × C), 128.58 (2 × C), 128.18, 127.86 (2 × C), 127.56, 127.03, 119.31, 118.75, 44.56, 25.73, 17.95. HRMS (ESI) *m*/*z* calculated for C_22_H_24_NO [M + H]^+^ 318.1858, found 318.1859. 

**3h**. 98 mg, 73% yield; light yellow oil. ^1^H NMR (400 MHz, CDCl_3_) δ: 7.71 (d, *J* = 15.6 Hz, 1H), 7.41–7.32 (m, 10H), 6.96 (d, *J* = 15.6 Hz, 1H), 6.61 (d, *J* = 8.8 Hz, 1H), 6.29 (d, *J* = 8.8 Hz, 1H), 4.23 (s, 2H), 4.18 (q, *J* = 7.2 Hz, 2H), 1.25 (t, *J* = 7.2 Hz, 3H). ^13^C NMR (100 MHz, CDCl_3_) δ: 169.02, 166.94, 144.01, 134.94, 134.02, 129.98, 128.79 (6 × C), 128.42, 128.10 (2 × C), 127.76, 125.69, 117.52, 61.30, 48.97, 14.16. HRMS (ESI) *m*/*z* calculated for C_21_H_22_NO_3_ [M + H]^+^ 336.1600, found 336.1606. 

**3i**. 115 mg, 85% yield; light yellow oil. ^1^H NMR (400 MHz, CDCl_3_) δ: 7.64 (d, *J* = 15.6 Hz, 1H), 7.35–7.23 (m, 15H), 6.92 (d, *J* = 15.6 Hz, 1H), 6.31 (d, *J* = 8.8 Hz, 1H), 6.28 (d, *J* = 8.8 Hz, 1H), 4.78 (s, 2H). ^13^C NMR (100 MHz, CDCl_3_) δ: 165.99, 143.12, 137.14, 135.20, 134.24, 129.10, 128.75 (4 × C), 128.70 (2 × C), 128.52 (4 × C), 128.34, 127.94 (2 × C), 127.51, 127.41, 127.26, 118.41, 50.03. HRMS (ESI) *m*/*z* calculated for C_24_H_22_NO_3_ [M + H]^+^ 340.1701, found 340.1728.

### 3.3. Bioassays

The antifungal activity of all synthesized compounds **1**–**3** was tested against ten plant pathogenic fungi, namely Phytophthora sojae, Sclerotinia sclerotiorum, Thanatephorus cucumeris, Fusahum graminearum, Pyricularia grisea, Fusarium solani, Rhizoctoniasolani Kuehn, Botrytis cinerea, Gloeosporium piperatum and Alternaria solani by the poison plate technique.

Compounds were dissolved in DMSO (10 mL) before mixing with Potato Dextrose Agar (PDA 90 mL) medium. The final concentration of compounds **1**–**3** in the medium were fixed at 100 µg/mL. Ten kinds of fungi were incubated in PDA at 25 °C for five days to get a new mycelium for the antifungal assay, then a mycelia disk of an approximately 0.45 cm diameter cut from the culture medium was picked up with a sterilized inoculation needle and inoculated in the center of the PDA plate. The inoculated plates were incubated at 25 °C for five days. DMSO in sterilized distilled water served as the control, while carbendazim was used as a positive control for each treatment, three replicates were carried out. The radial growth of the fungal colonies was measured on the sixth day and the data were statistically analyzed. The relative control efficacy of compounds compared to the blank assay was calculated via the following equation: I (%) = [(CK − PT/CK)] × 100%, where I is the relative control efficacy, CK is the average disease index during the blank assay, and PT is the average disease index after treatment during testing. The in vitro inhibiting effects of the test compounds on the fungi were calculated by the formula CV = (A − B)/A, where A represents the diameter of fungi growth on untreated PDA, B represents the diameter of fungi on treated PDA, and CV represents the rate of inhibition. All of the strains were conserved in the Key Laboratory of Biopesticide and Chemical Biology, Ministry of Education, Fujian Agriculture and Forestry University (Fuzhou, China).

## 4. Conclusions

In summary, we report the synthesis of a new series of Lansiumamide B derivatives in a simple and efficient way and the evaluation of their *in vitro* antifungal activity against ten plant pathogenic fungi. Most of the synthetic compounds exhibit a potential inhibition activity against all the tested fungi and some of them are more effective than carbendazim. Some of the compounds, especially **2** and **3a**, are identified as the most active and therefore of great potential to be developed as new antifungal agents. SAR analysis shows that the activity is highly sensitive to the substituent on the amide nitrogen. The introduction of methyl or ethyl group might enhance the activity, whereas the presence of N-H or group with larger steric hindrance causes decrease of activity. Therefore, these results are of great significance for the design, synthesis, and development of novel antifungal reagents containing Z-enamides. Further structural modification is ongoing in our laboratory, with the aim to secure compounds with improved antifungal activity.

## Figures and Tables

**Table 1:** Preliminary antifungal activities of all title compounds against five fungi at 100 µg/mL.

Compounds		Average Inhibition Rate ± SD (%) (n = 3)				
No.	R	*Thanatephorus cucumeris*	*Sclerotinia sclerotiorum*	*Botrytis cinerea*	*Phytophthora sojae*	*Fusahum graminearum*
**1**	H	53.89 ± 0.19	64.57 ± 0.50	27.09 ± 1.08	33.08 ± 0.61	18.09 ± 0.58
**2**	Me	91.20 ± 0.87	87.52 ± 2.49	80.60 ± 0.46	79.20 ± 1.76	76.63 ± 0.50
**3a**	Et	93.33 ± 0.66	66.55 ± 0.85	68.08 ± 1.14	62.16 ± 0.57	49.90 ± 0.55
**3b**	*n*-Pr	63.94 ± 1.14	58.23 ± 1.36	37.16 ± 0.96	49.41 ± 1.60	30.52 ± 1.21
**3c**	*n*-Bu	42.41 ± 0.95	43.61 ± 1.35	19.03 ± 0.76	36.57 ± 0.78	20.83 ± 1.12
**3d**	allyl	70.80 ± 0.94	62.45 ± 1.03	40.45 ± 0.85	60.19 ± 1.48	41.67 ± 1.82
**3e**	propargyl	64.28 ± 0.63	64.90 ± 1.17	77.70 ± 1.43	47.34 ± 0.57	62.04 ± 0.89
**3f**	2-methylallyl	47.13 ± 0.49	35.96 ± 0.95	29.50 ± 0.87	53.94 ± 0.78	17.08 ± 1.39
**3g**	prenyl	40.02 ± 1.58	26.98 ± 1.28	32.97 ± 1.98	19.47 ± 1.43	6.74 ± 0.44
**3h**	CH_2_CO_2_Et	27.34 ± 1.25	18.02 ± 0.37	27.23 ± 0.27	21.75 ± 0.62	26.95 ± 0.66
**3i**	benzyl	5.07 ± 0.81	25.00 ± 1.72	49.18 ± 0.93	59.01 ± 0.71	27.09 ± 0.76
Carbendazim		100	100	100	100	100

**Table 2:** Preliminary antifungal activities of all title compounds against another five fungi at 100 µg/mL.

Compounds		Average Inhibition Rate ± SD (%) (n = 3)				
No.	R	*Rhizoctoniasolani kuehn*	*Gloeosporium piperatum*	*Fusarium solani*	*Alternaria solani*	*Pyricularia grisea*
**1**	H	27.25 ± 0.37	18.43 ± 0.94	15.94 ± 0.72	33.72 ± 0.46	17.76 ± 0.83
**2**	Me	71.23 ± 1.20	66.21 ± 0.76	63.81 ± 0.23	52.52 ± 0.24	51.82 ± 1.30
**3a**	Et	49.81 ± 0.34	60.40 ± 1.47	67.40 ± 0.23	60.83 ± 0.35	45.32 ± 0.39
**3b**	*n*-Pr	47.97 ± 1.71	46.92 ± 0.56	38.86 ± 1.08	41.86 ± 0.99	43.03 ± 1.05
**3c**	*n*-Bu	33.99 ± 1.26	43.27 ± 1.24	33.14 ± 1.71	29.99 ± 0.35	11.50 ± 0.96
**3d**	allyl	48.98 ± 0.87	53.03 ± 0.60	52.29 ± 1.71	48.89 ± 0.68	30.67 ± 2.04
**3e**	propargyl	47.79 ± 0.71	65.22 ± 1.46	51.51 ± 0.55	55.69 ± 0.69	47.40 ± 0.29
**3f**	2-methylallyl	35.17 ± 1.72	47.56 ± 0.89	30.73 ± 1.05	35.06 ± 0.44	9.09 ± 1.44
**3g**	prenyl	31.87 ± 1.44	13.44 ± 0.27	27.30 ± 0.65	23.89 ± 1.26	15.50 ± 0.91
**3h**	CH_2_CO_2_Et	31.31 ± 2.02	25.38 ± 2.01	26.44 ± 0.55	45.50 ± 1.53	10.59 ± 0.97
**3i**	benzyl	20.82 ± 1.07	48.30 ± 1.21	19.37 ± 0.34	27.31 ± 1.15	32.94 ± 0.70
Carbendazim		100	100	100	1.25 ± 0.37	100

## Supplementary Materials

Copies of ^1^H, ^13^C, spectra of new compounds. This material is available free of charge online.
