# Supplementary material for: Synthesis and Fungicidal Activity of Lansiumamide A and B and Their Derivatives
Source: Molecules. 2018 Jun 21;23(7):1499. doi: 10.3390/molecules23071499 (PMC6099640; doi:10.3390/molecules23071499)

***Supporting Information***

Synthesis and Fungicidal Activity of Lansiumamide A and B and Their Derivatives

Huiyou Xu, Ting Chen, Zengwei Lian, Yan Shi, Ming-An Ouyang and Liyan Song*

Key Laboratory of Biopesticide and Chemical Biology, Ministry of Education, Fujian Agriculture and Forestry University, Fuzhou, Fujian, China;

*** s**ongliyan@fafu.edu.cn

**Table of Content**

| **^1^H NMR and ^13^C NMR spectra of all compounds in CDCl_3_.** | **S-2** |
| --- | --- |


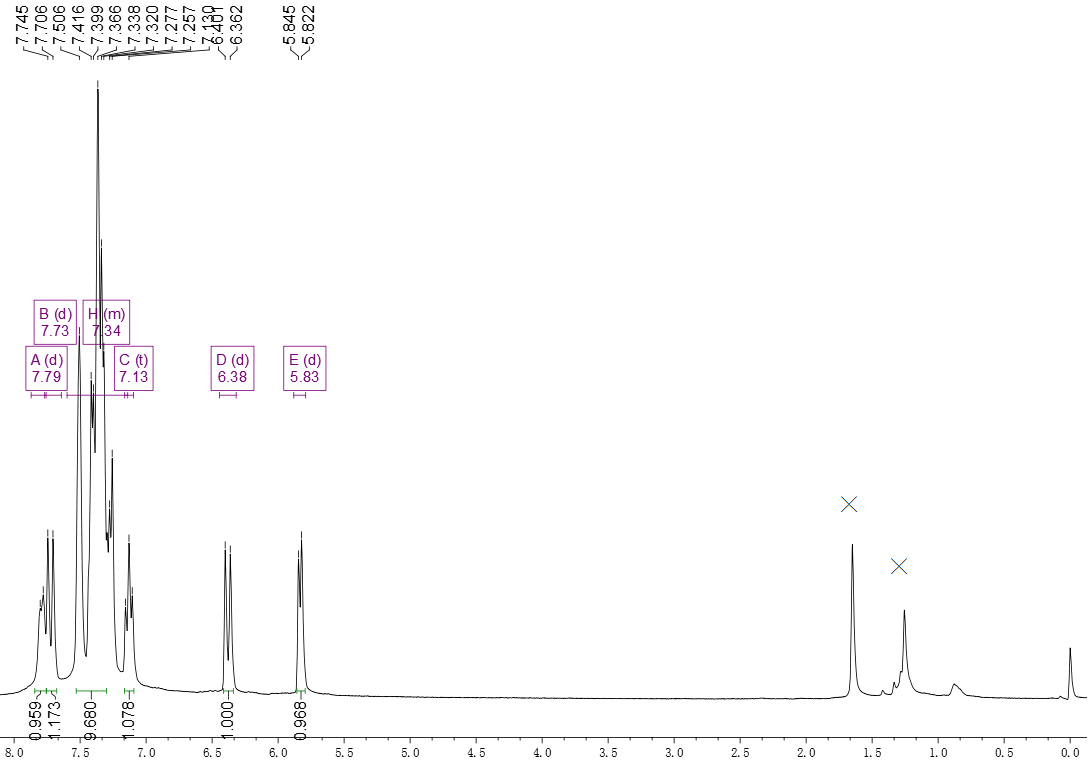


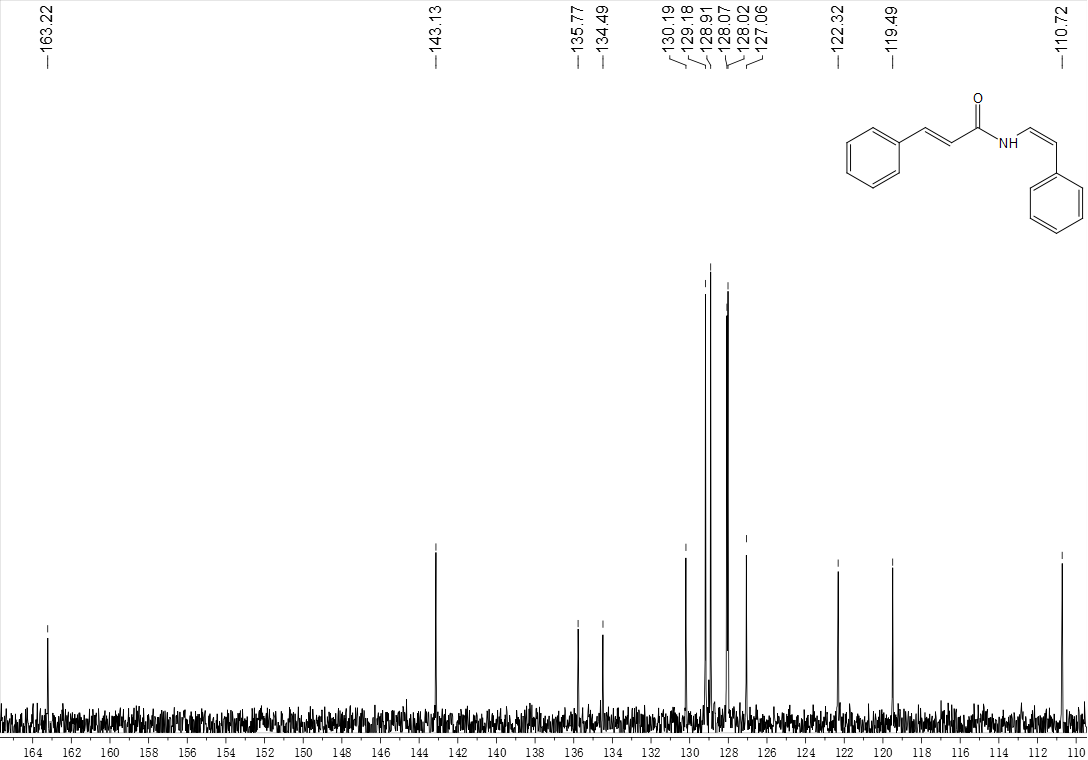


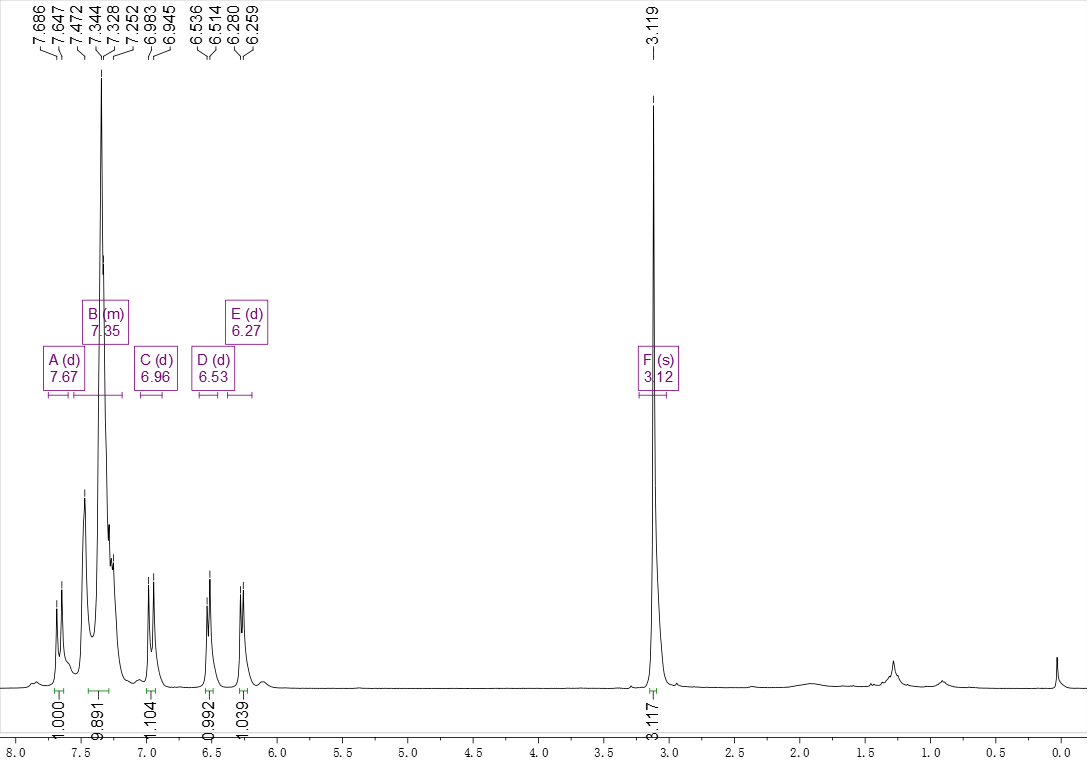


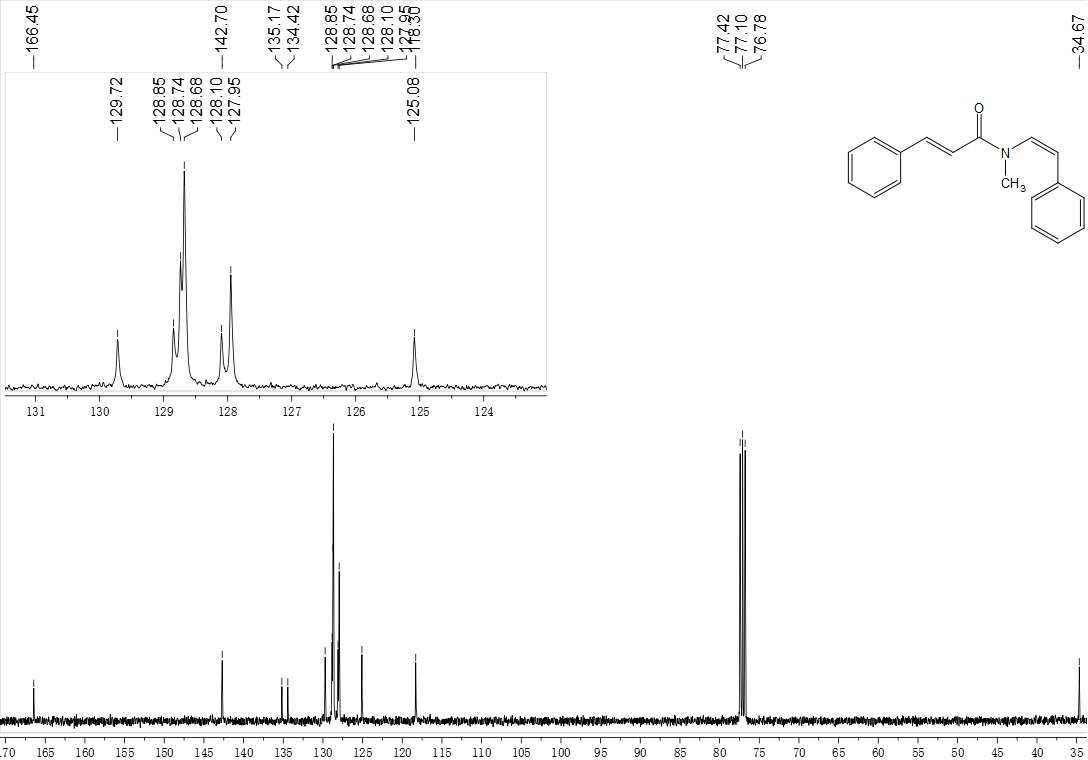


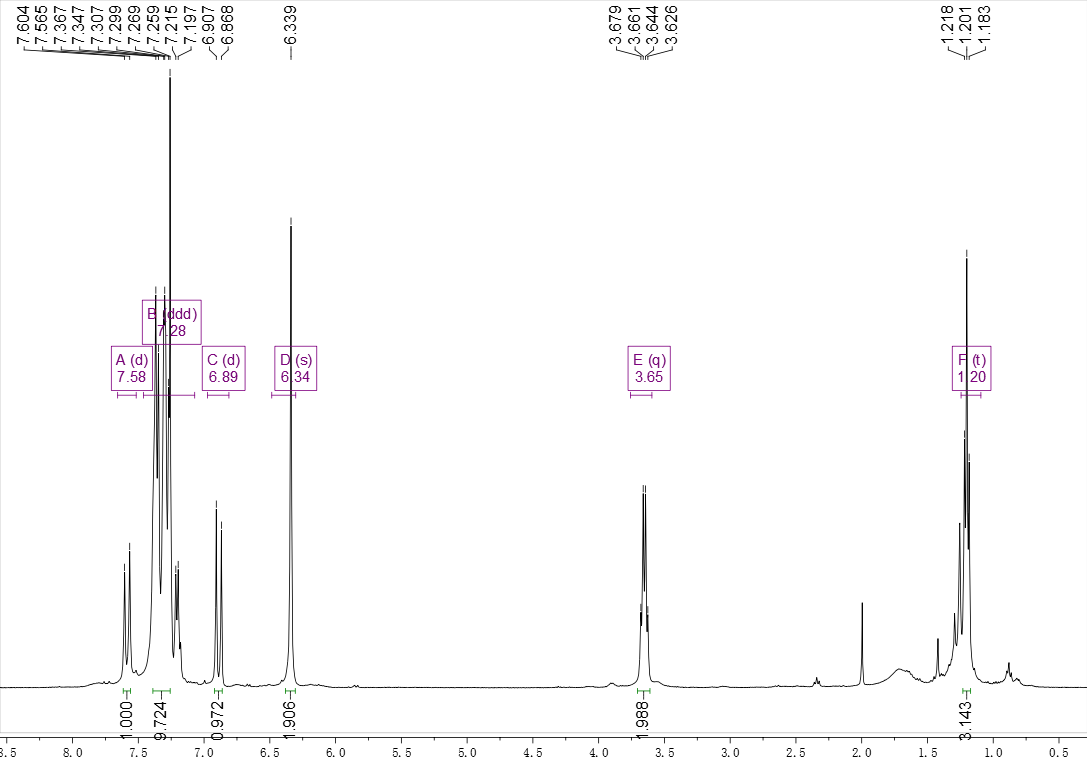


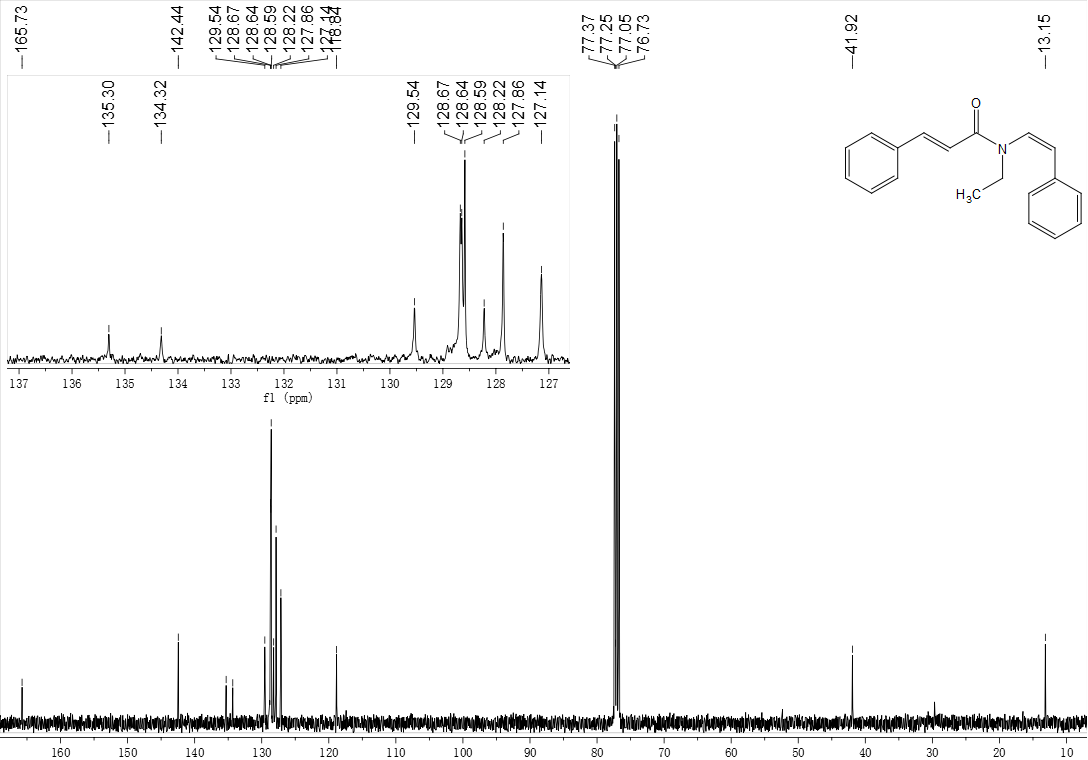


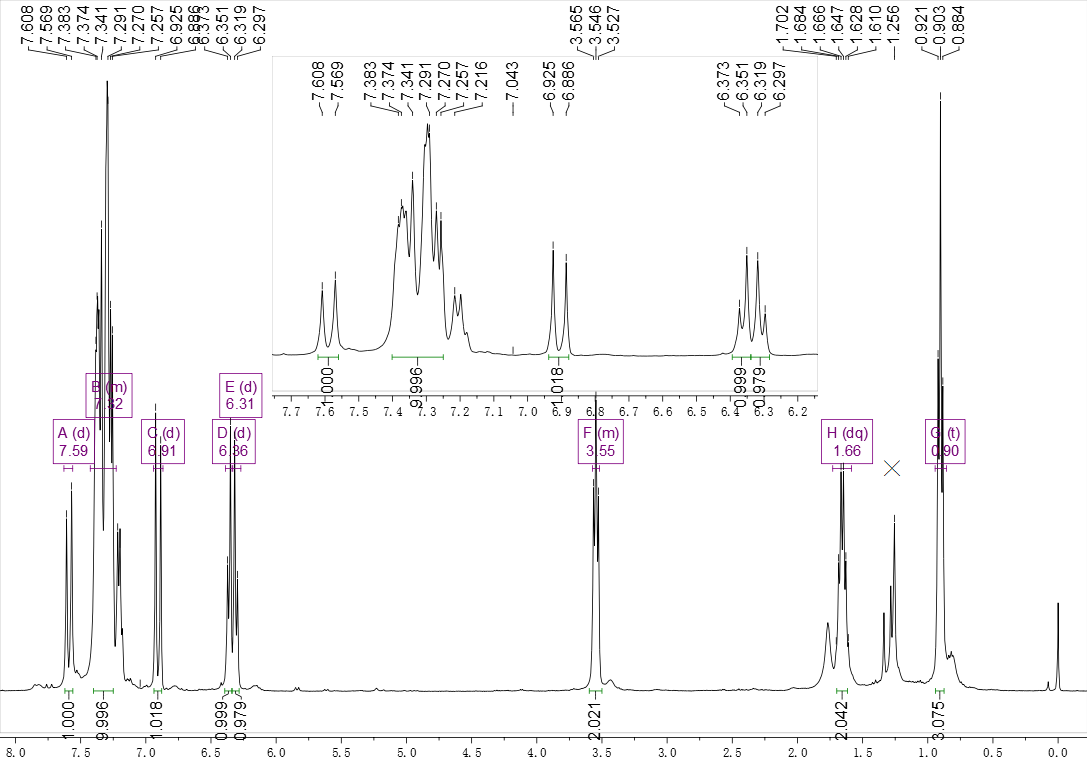


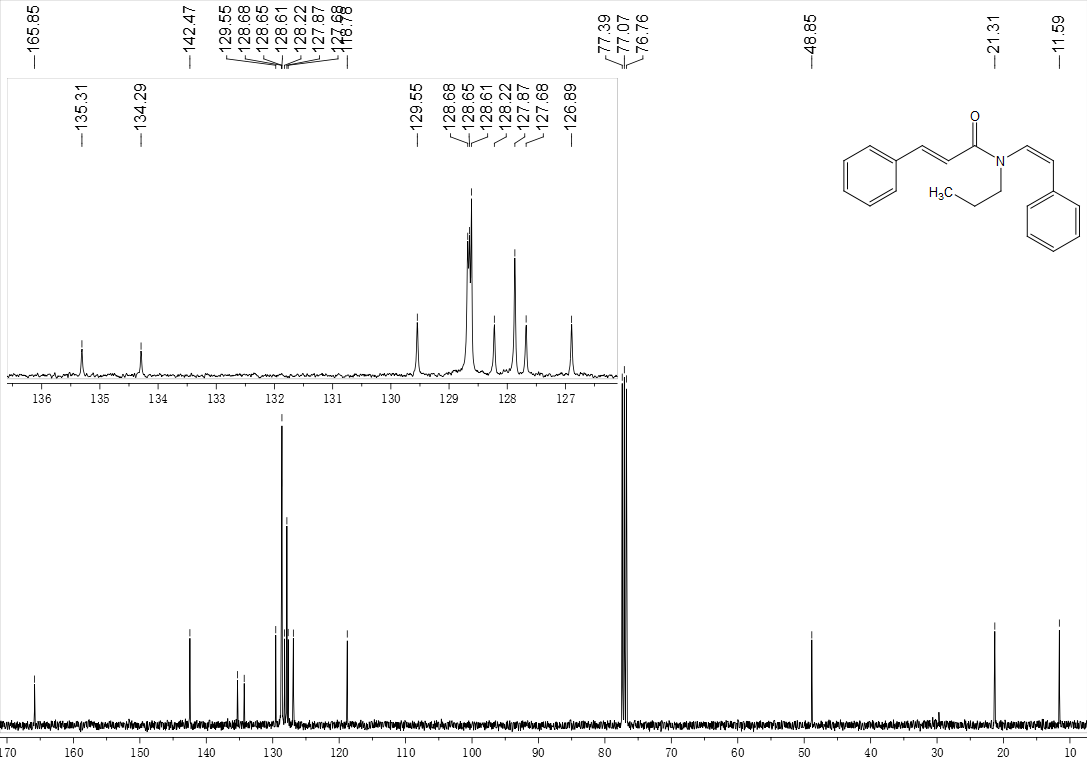


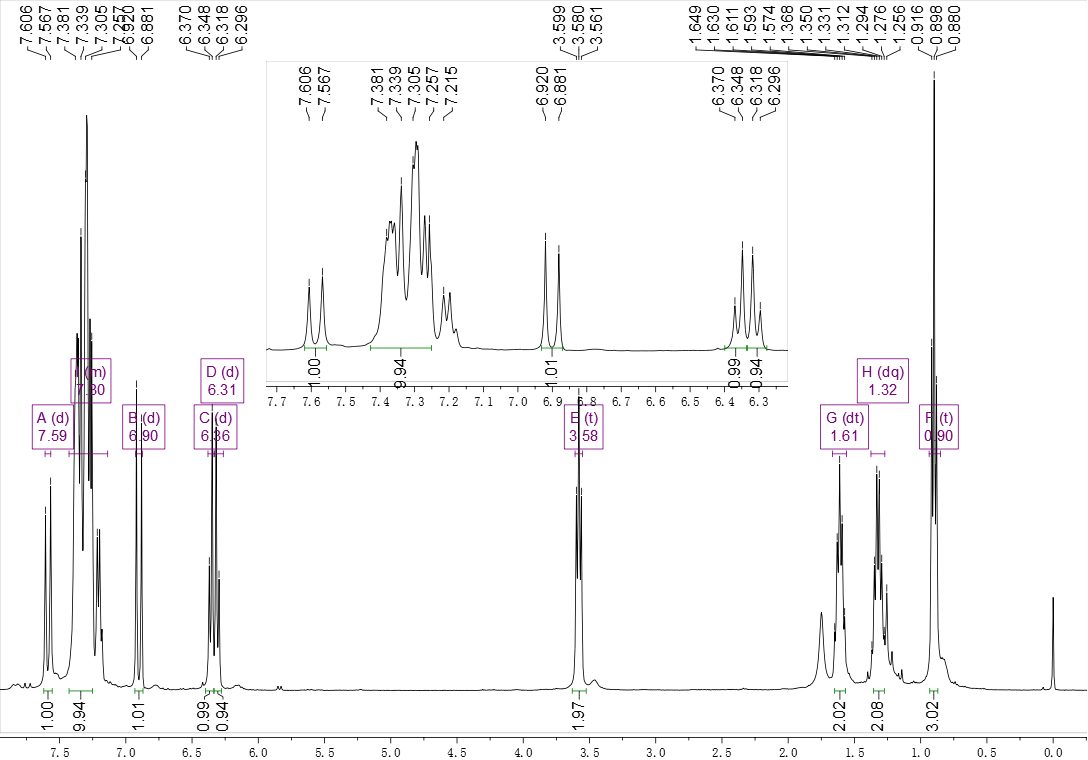


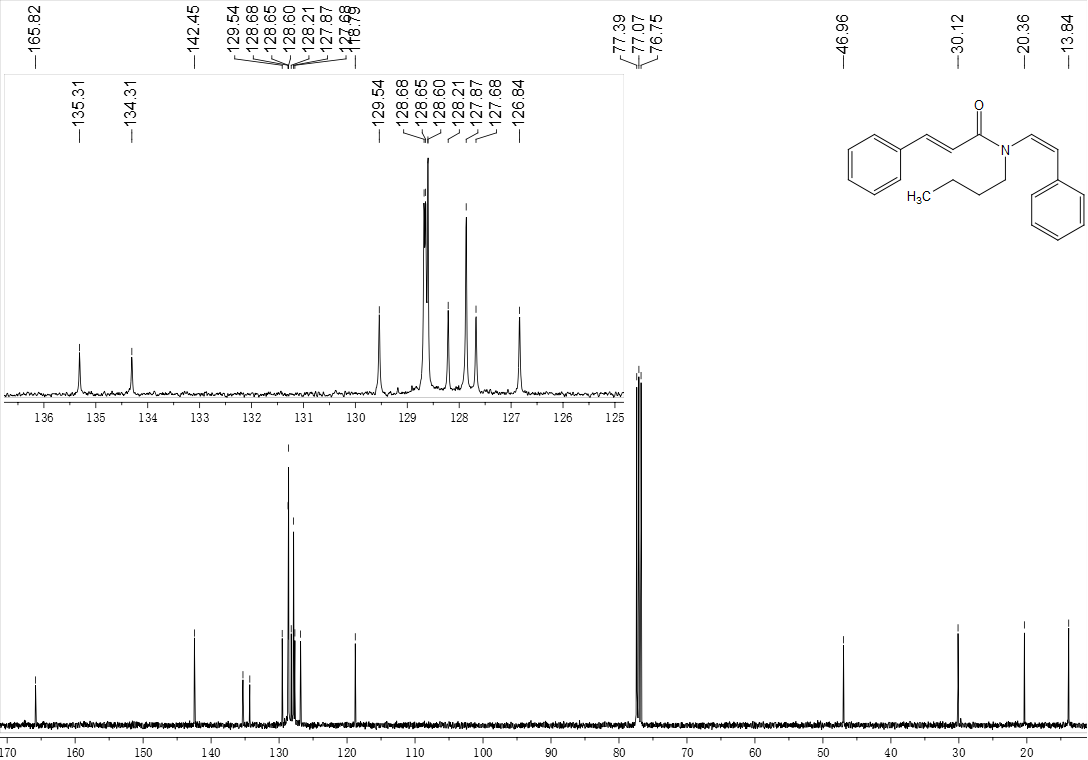


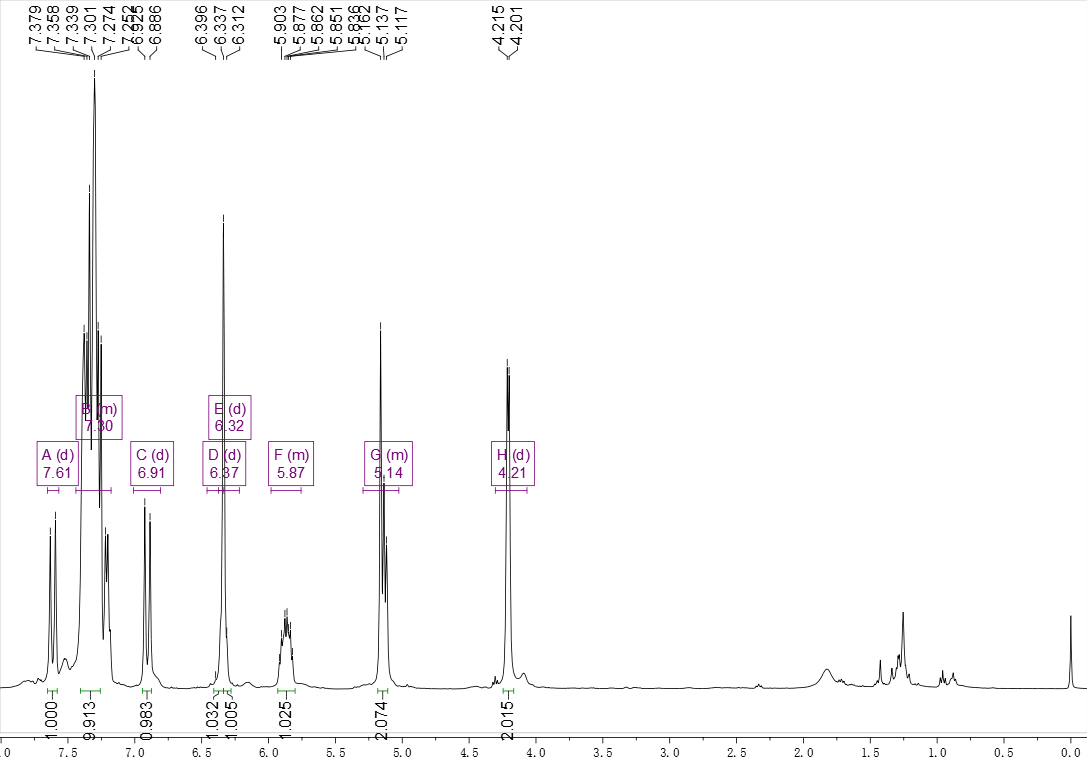


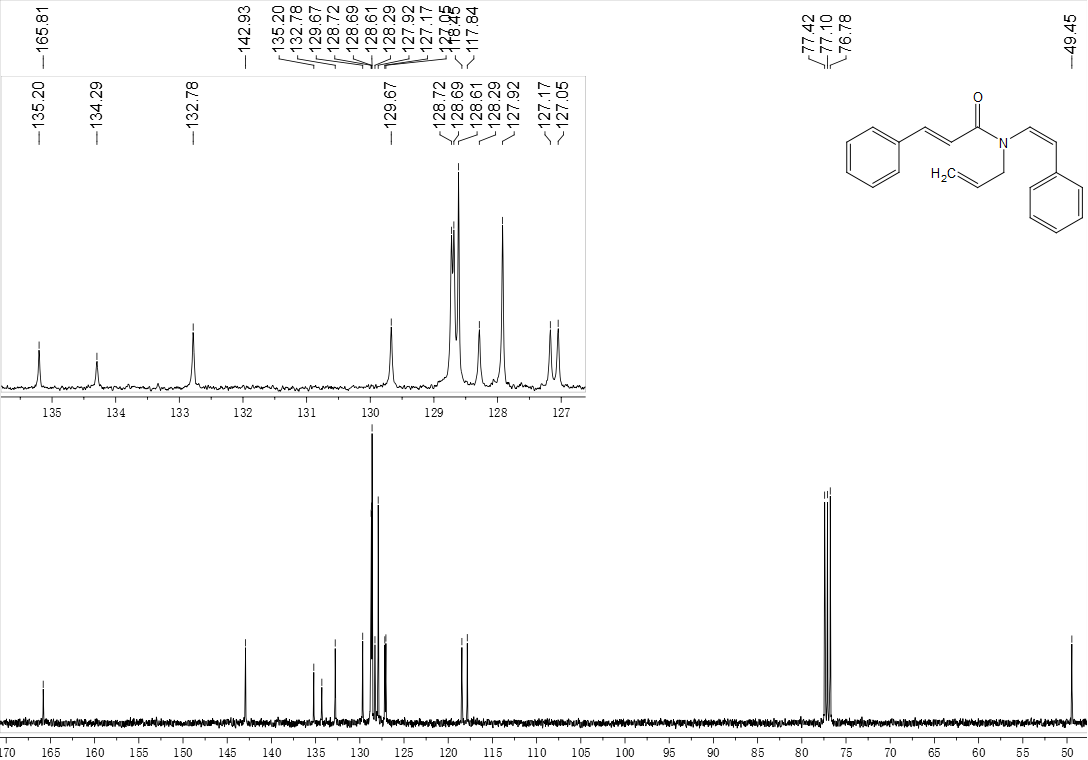


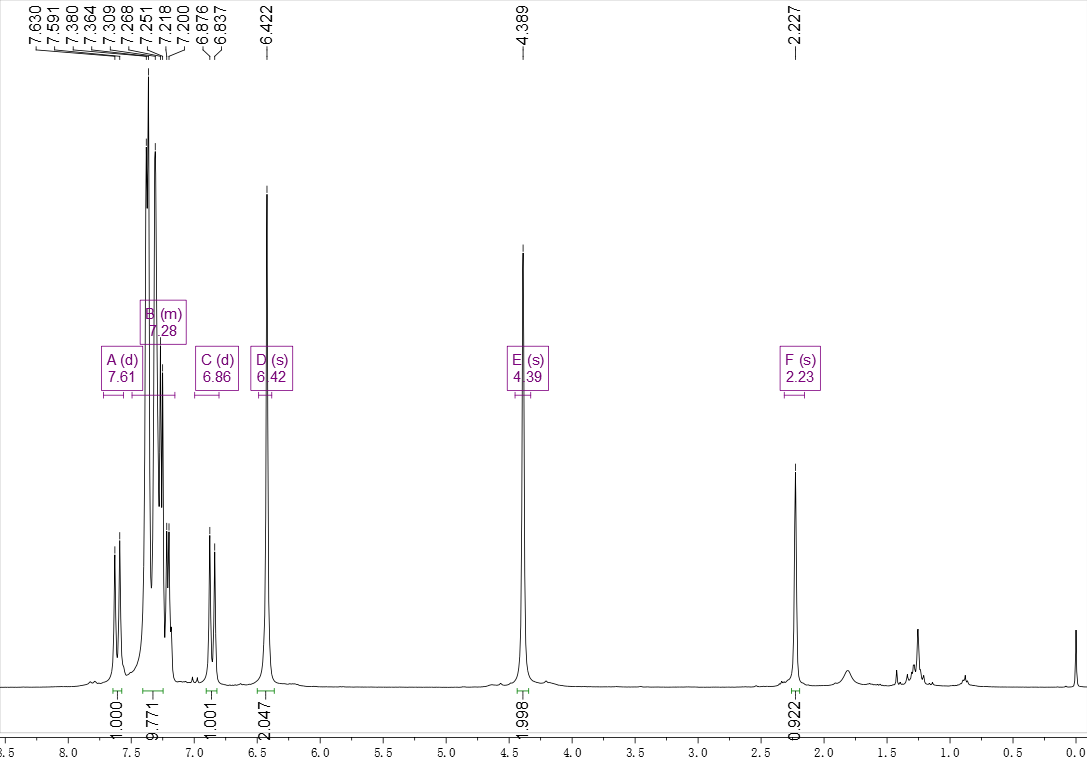


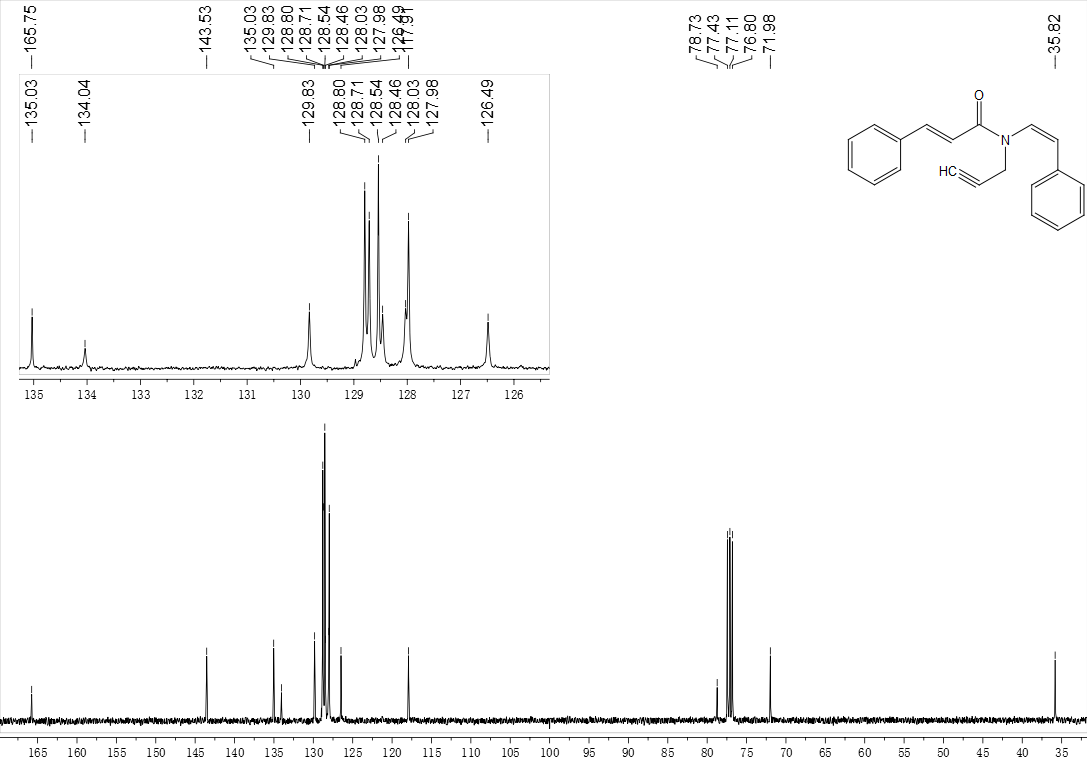


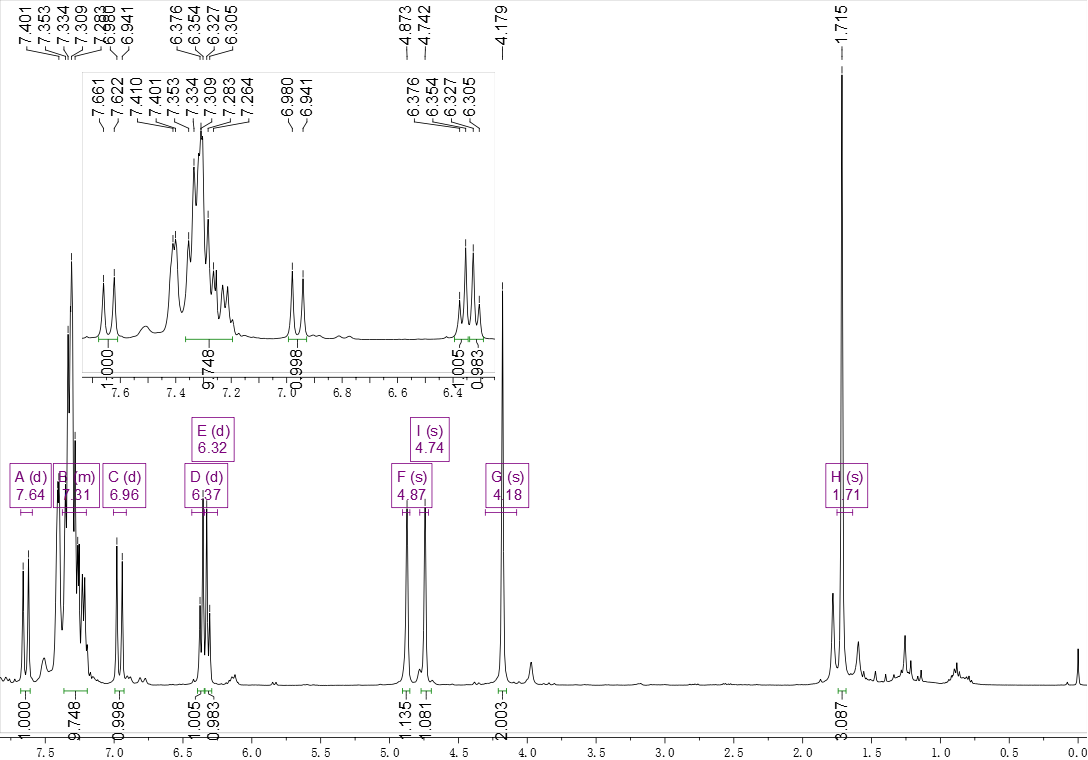


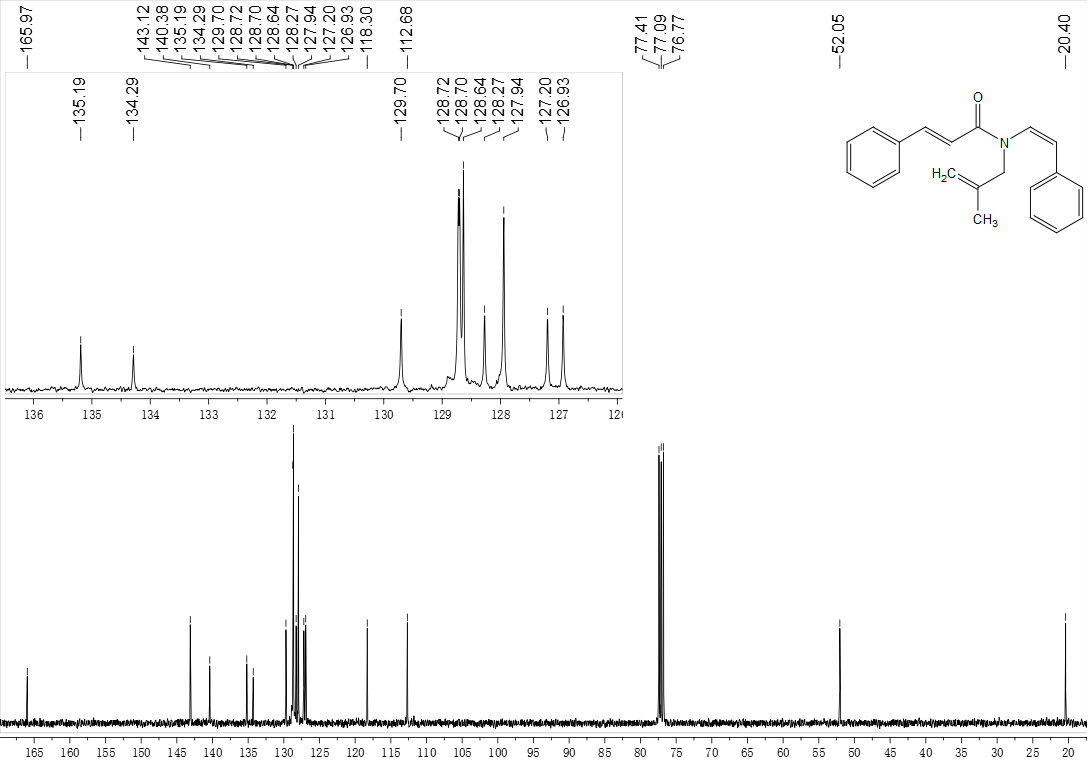


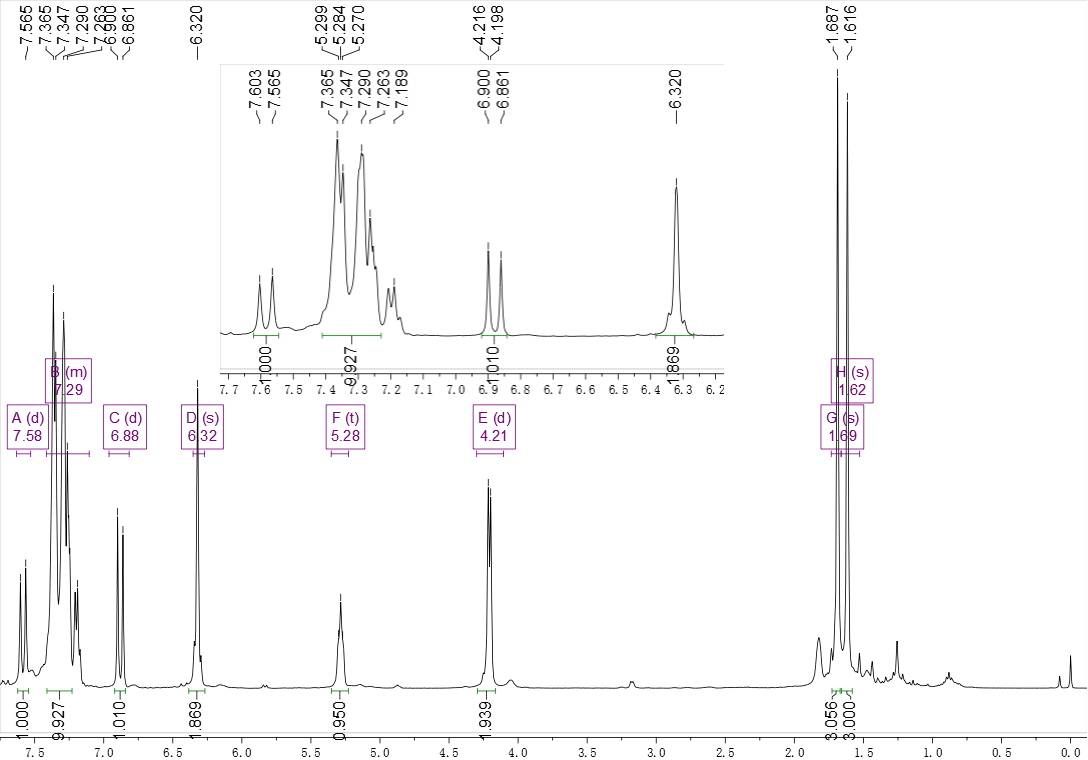


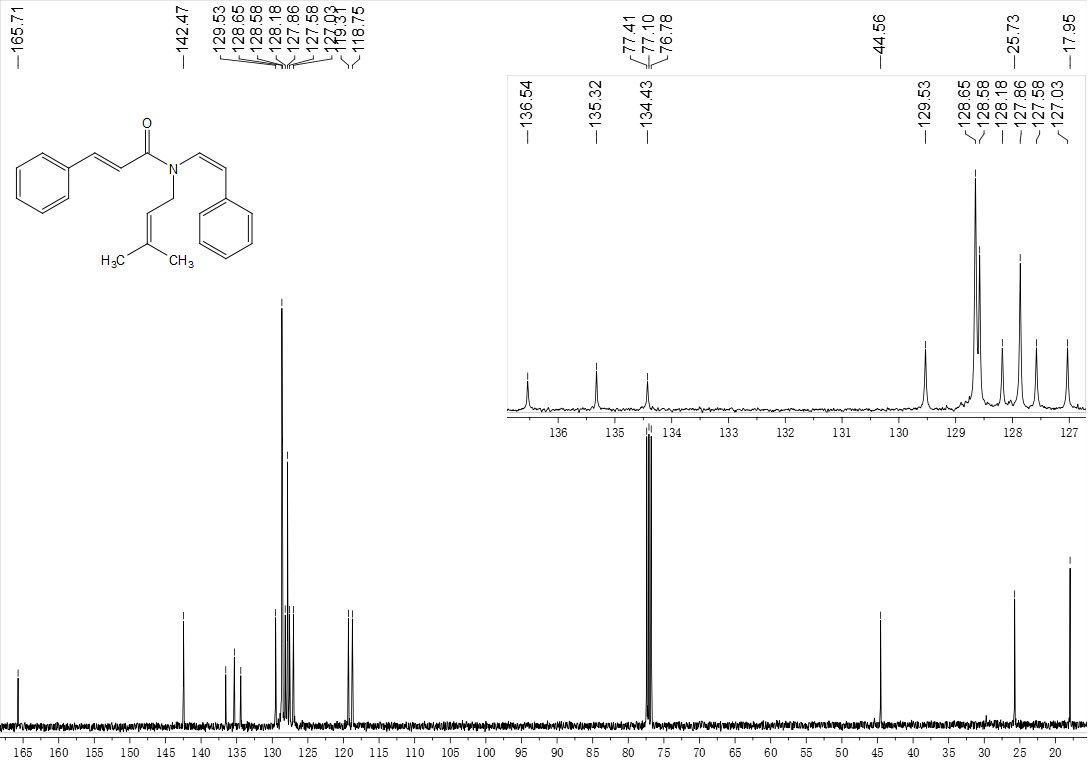


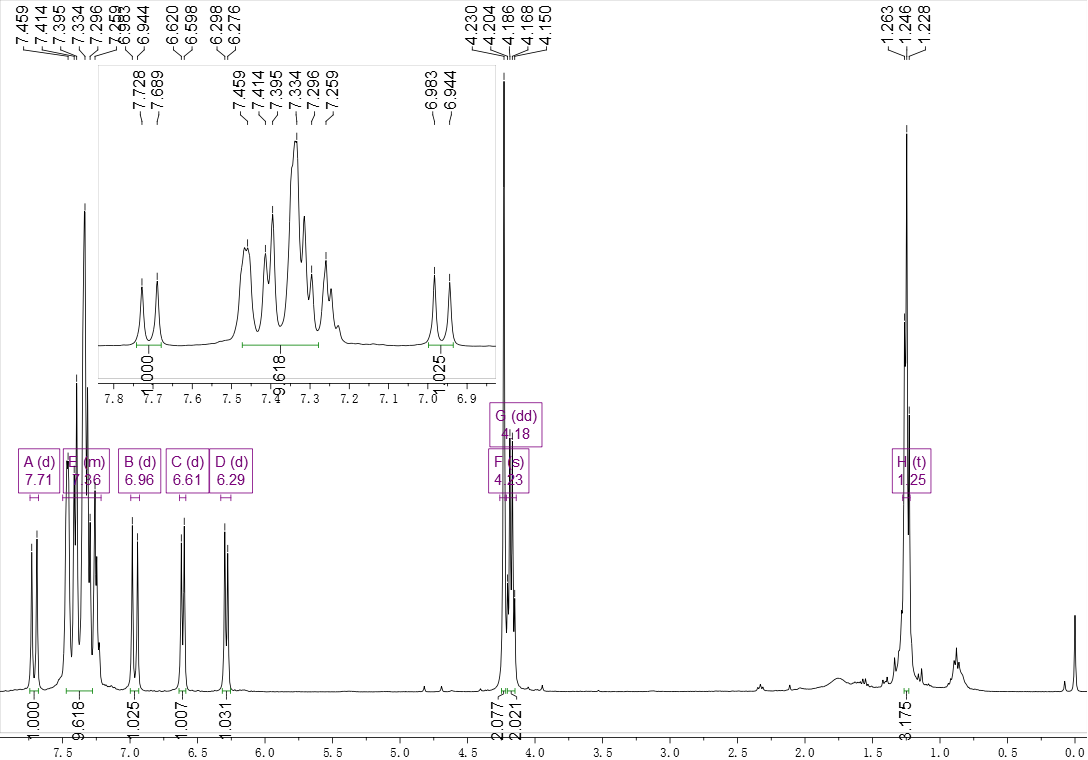


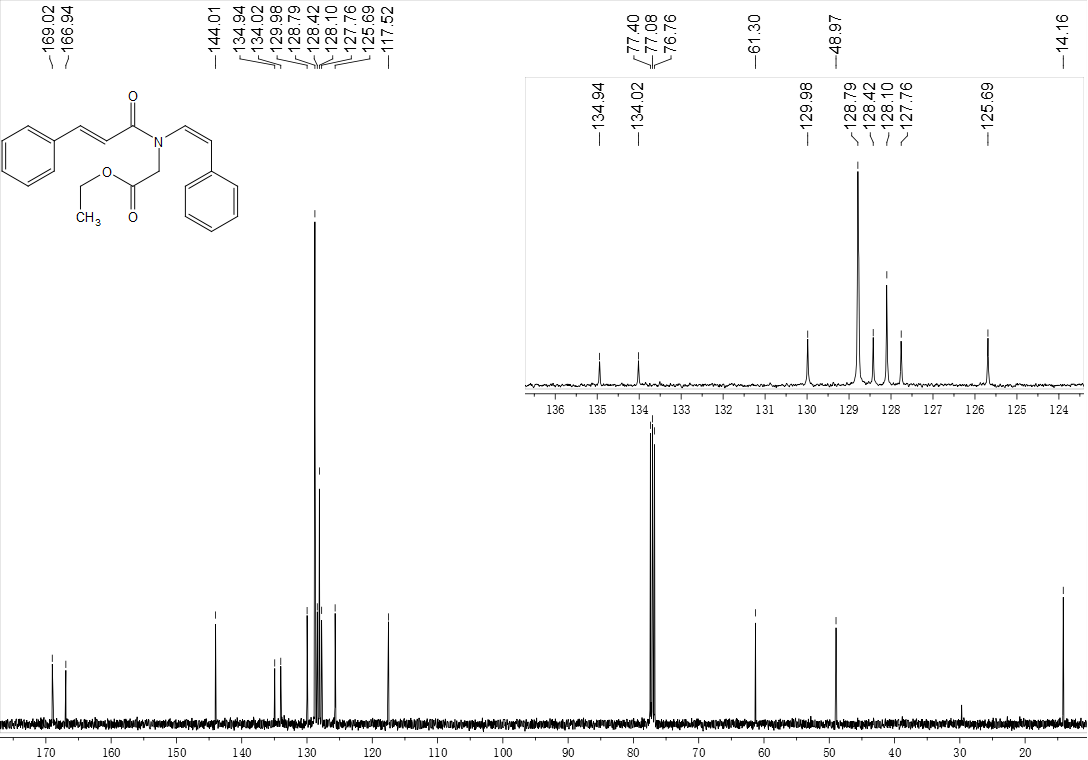


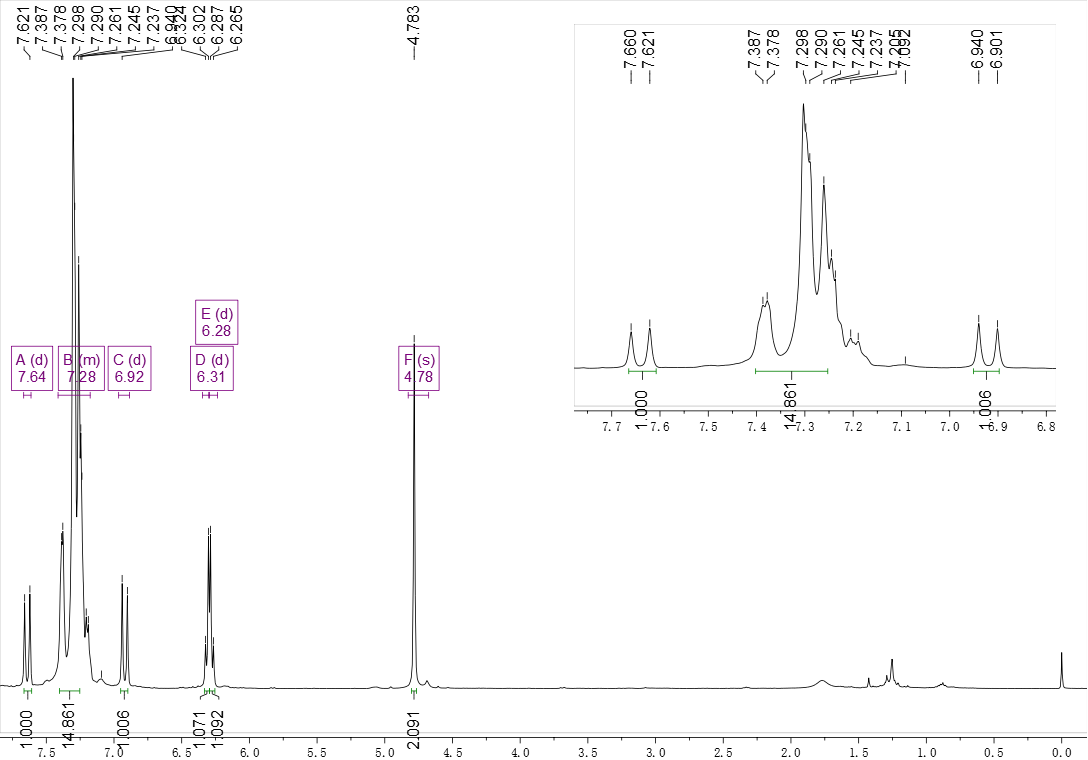


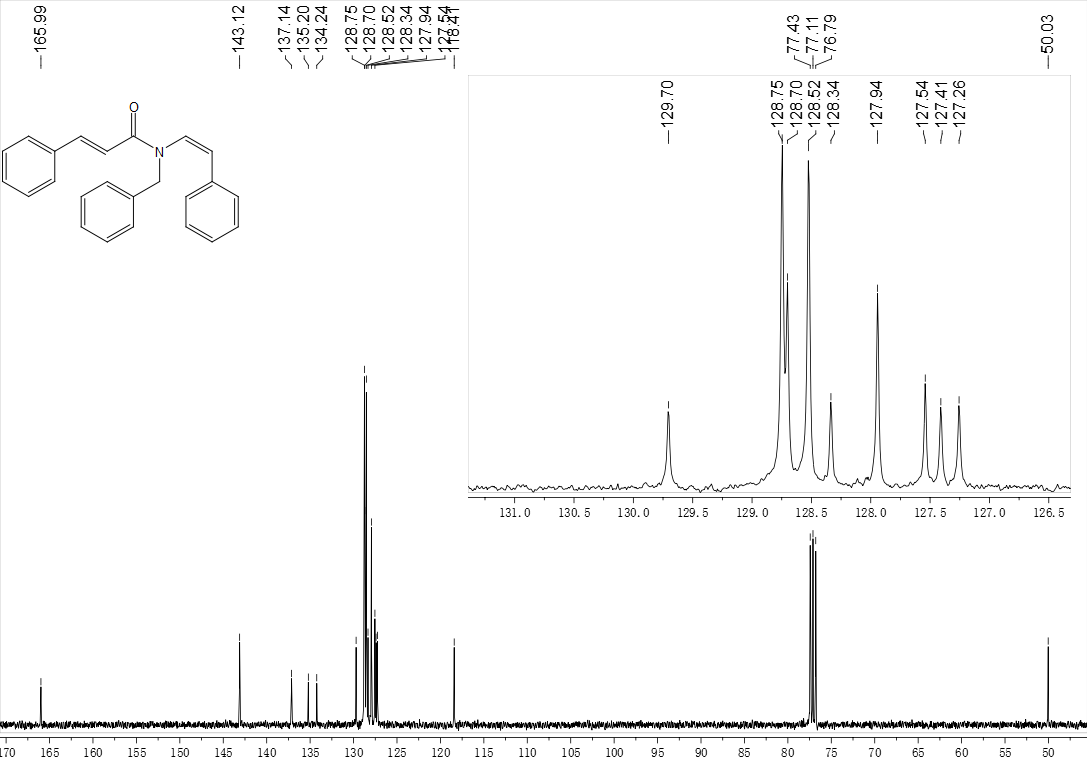

Supplement: Supplementary file 1 [file molecules-23-01499-s001.docx]
